# The independent and joint relationships between dietary antioxidant intake with risk of chronic obstructive pulmonary disease and all-cause mortality: insights from NHANES

**DOI:** 10.3389/fpubh.2024.1393119

**Published:** 2025-01-29

**Authors:** Yifeng Xu, Zhaoqi Yan, Keke Li, Liangji Liu, Lei Xu

**Affiliations:** ^1^School of Clinical Medicine, Jiangxi University of Chinese Medicine, Nanchang, Jiangxi, China; ^2^Guang’anmen Hospital, China Academy of Chinese Medical Sciences, Beijing, China; ^3^Affiliated Hospital of Jiangxi University of Traditional Chinese Medicine, Nanchang, Jiangxi, China

**Keywords:** comprehensive dietary antioxidant index, chronic obstructive pulmonary disease, NHANES, oxidative stress, restricted cubic spline

## Abstract

**Background:**

Numerous studies have indicated that a diet rich in antioxidants can prevent Chronic Obstructive Pulmonary Disease (COPD). However, the relationship between combined dietary antioxidant intake and the risk of COPD remains unclear. The Comprehensive Dietary Antioxidant Index (CDAI) is a composite score of various dietary antioxidants, including vitamins A, C, E, zinc, selenium, and carotenoids. In this study, we examined the independent and joint associations of CDAI with the risk of COPD and all-cause mortality.

**Methods:**

The analysis was conducted using data from the National Health and Nutrition Examination Survey (NHANES) spanning from 2013 to 2018. Multivariable weighted logistic and Cox regression models were employed to evaluate the correlations of CDAI (including vitamins A, C, E, zinc, selenium, and carotenoids) with the risk of COPD and all-cause mortality. Restricted cubic spline models were used to examine potential non-linear relationships. Sensitivity and stratified analyses were conducted to assess the robustness of the results.

**Results:**

In this study, compared to the lowest quartile, the highest quartile of CDAI levels was significantly negatively associated with the risk of COPD (Odds Ratio [OR]: 0.47; 95% Confidence Interval [CI]: 0.22–0.97), exhibiting a non-linear relationship. Additionally, vitamins A, C, E, zinc, selenium, and carotenoids were also negatively associated with the risk of COPD. Furthermore, dietary antioxidant zinc in the second quartile (Hazard Ratio [HR]: 0.25; 95% CI: 0.10–0.62) was negatively associated with the risk of all-cause mortality compared to the lowest quartile. Subgroup analysis results remained stable, and sensitivity analysis did not support the association between selenium and the risk of COPD, with no substantial changes in the remaining associations.

**Conclusion:**

Higher CDAI is inversely associated with the risk of COPD, and appropriate intake of the dietary antioxidant zinc may reduce all-cause mortality in COPD patients.

## Introduction

Chronic Obstructive Pulmonary Disease (COPD) is a major contributor to the global burden of healthcare systems, affecting approximately 10% of the population aged 40 and above. It is projected that by 2030, COPD may become the third leading cause of death worldwide (1). The condition is characterized by persistent and usually progressive airflow obstruction due to abnormalities in the airways and/or alveoli, leading to chronic respiratory symptoms such as dyspnea, cough, and sputum production (2). Increasing evidence suggests that pulmonary oxidative stress is a primary driving mechanism of COPD (3). Compared to healthy controls, markers of systemic and airway oxidative damage (related to deoxyribonucleic acid, proteins, and lipids) are often elevated in individuals with COPD (4). Oxidative stress occurs due to the presence of reactive oxygen species (ROS), leading to compromised and/or overwhelmed endogenous antioxidant defenses. This indicates that reducing oxidative stress through targeted antioxidant interventions or enhancing endogenous antioxidant levels may be a useful therapeutic approach (5).

Diet is considered a major modifiable factor in the development and progression of chronic diseases (6), and many dietary components possess anti-inflammatory and antioxidant properties. Research has indicated a negative correlation between serum 25-(OH)-D concentration and the incidence and mortality of COPD, suggesting a potential protective role of vitamin D in the pathogenesis of COPD (7). In a cross-sectional study involving a Korean population, Hye Jung Park demonstrated that dietary vitamin C may prevent chronic obstructive pulmonary disease (8). It is noteworthy that most studies have focused on the impact of individual food categories or antioxidants on COPD, which may not adequately reflect overall antioxidant intake. Current dietary guidelines emphasize holistic dietary patterns (6), such as the Healthy Eating Index (9) and the Dietary Approaches to Stop Hypertension diet pattern (10). However, assessing only overall dietary patterns may obscure the influence of individual foods. Therefore, the impact of dietary intake should consider both independent and overall associations.

The Comprehensive Dietary Antioxidant Index (CDAI) is a composite score of various dietary antioxidants (11), representing the overall antioxidant characteristics of an individual’s diet, including vitamin A, vitamin C, vitamin E, zinc, selenium, and carotenoids. Previous studies have indicated that a high CDAI may reduce the risk of colorectal cancer, diabetes, heart failure, and all-cause mortality in patients with osteoarthritis (12–15). However, within the US population, there is limited research on the relationship between the CDAI and COPD. Therefore, based on the National Health and Nutrition Examination Survey (NHANES) database, we assessed the associations of CDAI (including vitamin A, vitamin C, vitamin E, zinc, selenium, and carotenoids) with the risk of COPD and all-cause mortality.

## Methods

### Study population

For this cross-sectional study, we obtained data from three NHANES cycles (2013–2014, 2015–2016, 2017–2018) as the basis for analysis. NHANES, led by the Centers for Disease Control and Prevention (CDC), is a nationally representative survey aimed at assessing the health and nutritional status of both adults and children in the United States. It includes demographic, dietary, examination, laboratory, and questionnaire data. Currently, data are collected and released every 2 years by the National Center for Health Statistics (NCHS) at the CDC. The NHANES study protocol has obtained approval from the Research Ethics Review Board of the NCHS, and written informed consent has been obtained from all participants.

**Figure 1:**
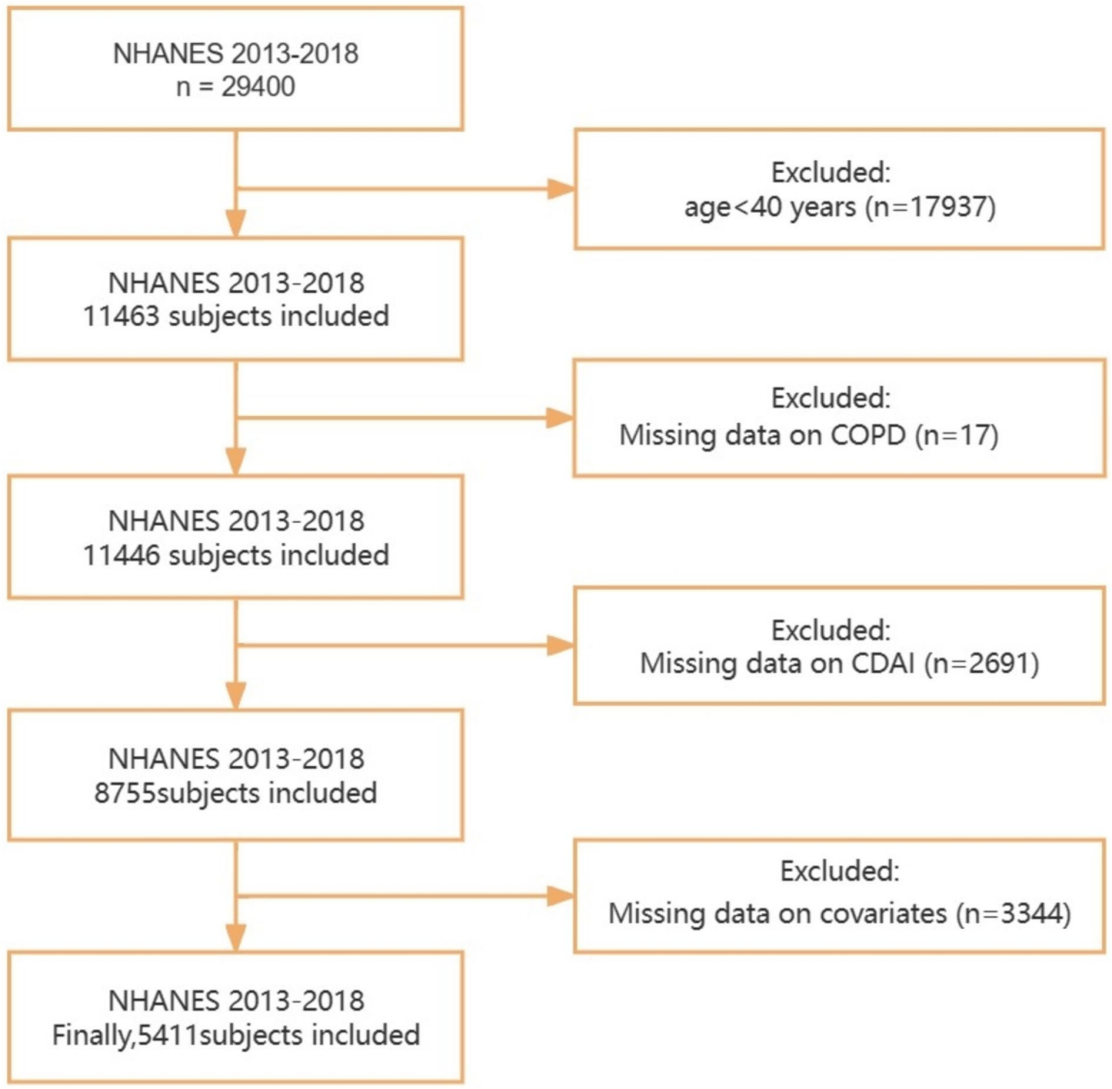
Flowchart for the selection of eligible participants. NHANES, National Health and Nutrition Examination Survey; CDAI, Composite Dietary Antioxidant Index; COPD, Chronic obstructive pulmonary disease.

The exclusion criteria for this study are as follows (Figure 1): (1) individuals below the age of 40; (2) individuals with missing COPD data; (3) individuals with incomplete or unavailable CDAI data; (4) individuals with incomplete data on covariates such as smoking status, body mass index (BMI), hypertension, total calories, diabetes, etc. Additionally, individuals with implausible total calories intake were excluded (males <600 or > 4,200 kcal, females <500 or > 3,600 kcal) (16). Finally, a total of 5,411 individuals met the inclusion criteria for this study.

### Exposure variable

In the NHANES survey, information on dietary antioxidant intake and other food components was obtained through two separate 24-h dietary recall interviews, and the average of the two recalls was used for assessment. Participants were asked to recall the details of food and beverage consumption in the 24 h preceding each interview. The first recall was collected in person at the Mobile Examination Center, while the second recall was conducted via telephone 3–10 days later. The United States Department of Agriculture Food and Nutrient Database for Dietary Studies was used to calculate the intake of antioxidants, micronutrients, and total energy (17). We investigated six dietary antioxidant exposures: vitamin A, vitamin C, vitamin E, zinc, selenium, and carotenoids. To assess the comprehensive exposure of dietary antioxidant intake, we utilized a modified version of the CDAI developed by Wright et al (11). We calculated the CDAI by subtracting the mean from the intake of each antioxidant and dividing the result by the standard deviation (SD), and finally adding the standardized intake of these dietary antioxidants.


$$\mathrm{CDAI}=\overset{n=6}{\underset{i=1}{\sum }}\frac{\mathrm{Individual Intake}-\mathrm{Mean}}{SD}$$

### Outcome

The definition of COPD is a positive response to the question: “Has a doctor or other health professional ever told you that you have COPD?” Previous research utilizing NHANES data has indicated that self-reported COPD diagnosis is an effective method (18–20).

### Identification of mortality

To assess the association of CDAI (including vitamin A, vitamin C, vitamin E, zinc, selenium, and carotenoids) with all-cause mortality in COPD patients, we further conducted a cohort analysis. All-cause mortality was determined using mortality data recorded by the National Death Index (NDI) up to December 31, 2019. These records are linked to NHANES data, and mortality archives are available online.^1^ The defined cause of death is determined based on the International Classification of Diseases, 10th Revision (ICD-10).

### Covariates definition

The covariates in this study include age, gender, ethnicity (Non-Hispanic White, Non-Hispanic Black, Mexican American, Other race), education level (Less than high school, High School Grad/GED, More than high school), marital status (Married/cohabiting, Never married, Widowed/divorced/separated), body mass index (BMI), poverty income ratio (PIR), smoking status, Physical activity, total calories, hypertension, diabetes, and cardiovascular disease (CVD). BMI is categorized as <25 (kg/m^2^), ≥25 (kg/m^2^), and PIR as <1.85, ≥1.85. Smoking status is classified as smoking or never smoking; smokers are defined as those who have smoked more than 100 cigarettes, and never smokers are defined as those who have smoked fewer than 100 cigarettes in their lifetime. Physical activity was converted to metabolic equivalent (MET) scores, with MET minutes/week ≥500 considered active and MET minutes/week <500 deemed inactive. For CVD, a positive response to whether a doctor or other health professional has ever told you that you have congestive heart failure (CHF)/coronary heart disease (CHD)/angina/heart attack/stroke was defined as having CVD. Hypertension is defined as a self-reported diagnosis of high blood pressure or with antihypertensive medication administration. Diabetes is based on self-reported diagnosis and use of diabetes medication.

### Statistical analyses

The study participants’ characteristics were reported for continuous variables as mean ± SD and for categorical variables as percentages. Participants were divided into two groups based on whether they were COPD patients, and differences between COPD and non-COPD subjects were assessed using weighted t-tests (for continuous variables) and weighted chi-square tests (for categorical variables). Additionally, covariates were incorporated into nomogram to predict the risk of COPD. The accuracy of the nomogram was assessed using receiver operating characteristic (ROC) curve, and its discriminative ability was verified through calibration plot. Decision curve analysis (DCA) was conducted to evaluate the clinical utility of different models (21).

Multivariate logistic regression analysis was employed to assess the odds ratios (ORs) and 95% confidence intervals (CIs) for the association between CDAI and the intake of six different dietary antioxidants with the risk of COPD. Furthermore, multivariate Cox regression analysis was conducted to evaluate the hazard ratios (HRs) and 95% CIs for the association between CDAI, the intake of six dietary antioxidants, and all-cause mortality in COPD patients. Regression models were tested by stepwise adjustment of covariates (Models 1 to 3): Model 1 adjusted for age, sex, and ethnicity; Model 2 further adjusted for PIR, BMI, education level, smoking status, and physical activity; Model 3 additionally adjusted for cardiovascular disease, hypertension, total calories and diabetes. Restricted cubic splines (RCS) were employed to assess the non-linear association between CDAI and the dietary intake of vitamin A, vitamin C, vitamin E, zinc, selenium, and carotenoids with the risk of COPD and all-cause mortality at four knots (5th, 35th, 65th, and 95th percentiles). Stratified analyses were performed based on age (<60 or ≥ 60 years), sex (male or female), smoking status (never smoker, smoker), BMI (<25 or ≥ 25), hypertension (yes or no), CVD (yes or no), and diabetes (yes or no), using interaction terms to assess potential interaction effects between these variables.

A series of sensitivity analyses were conducted: (1) excluding participants who died within 2 years of follow-up to eliminate potential reverse causation; (2) repeating the main analysis based on the quintiles of CDAI and the six dietary antioxidants; (3) Considering the impact of early life events on COPD, we conducted a re-analysis with additional adjustments for childhood asthma. Statistical analyses were conducted using R Studio (version 4.2.2), and the significance level was set at *p* < 0.05 (two-sided).

## Results

### General characteristics of the study population

The study included a total of 5,411 participants (mean [SD] age of 58 [11] years), among whom 272 were diagnosed with COPD (154 males [weighted proportion 49%], 118 females [weighted proportion 51%]). Compared to the Non-COPD group, COPD patients were older (64 [10] years vs. 57 [11] years), had a higher proportion of non-Hispanic White individuals (192 [84%] vs. 2,116 [73%]), were more economically disadvantaged (161 [46%] vs. 1,878 [21%]), had a higher percentage of smokers (233 [87%] vs. 2,276 [44%]), and were more likely to have comorbidities such as CVD (109 [38%] vs. 613 [9.3%]), hypertension (173 [60%] vs. 2,304 [39%]), and diabetes (76 [28%] vs. 933 [13%]). Additionally, there were statistically significant differences between the two groups in terms of education level and marital status (all *p*-values <0.05; Table 1).

**Table 1:** Baseline characteristics of study participants by incident COPD.

Characteristic	Overall, *N* = 5,411 (100%)^1^	COPD, *N* = 272 (5%)^1^	Non-COPD, *N* = 5,139 (95%)^1^	*p* value^2^
Age (years)	58 (11)	64 (10)	57 (11)	**<0.001**
Sex				0.8
Female	2,664 (50%)	118 (51%)	2,546 (50%)	
Male	2,747 (50%)	154 (49%)	2,593 (50%)	
Ethnicity				**0.003**
Non-Hispanic White	2,308 (74.1%)	192 (84%)	2,116 (73%)	
Other Race	1,271 (11%)	34 (10%)	1,237 (11.5%)	
Non-Hispanic Black	1,172 (9.3%)	39 (5.3%)	1,133 (9.6%)	
Mexican American	660 (5.6%)	7 (0.7%)	653 (5.9%)	
PIR				**<0.001**
<1.85	2,039 (23%)	161 (46%)	1,878 (21%)	
≥1.85	3,372 (77%)	111 (54%)	3,261 (79%)	
BMI (kg/m^2^)				0.9
<25	1,341 (25%)	66 (25%)	1,275 (25%)	
≥25	4,070 (75%)	206 (75%)	3,864 (75%)	
Smoking status				**<0.001**
Smoker	2,509 (46%)	233 (87%)	2,276 (44%)	
Never smoker	2,902 (54%)	39 (13%)	2,863 (56%)	
Education attainment				**<0.001**
High School Grad/GED	1,231 (22.7%)	94 (40%)	1,137 (21.6%)	
Less than high school	928 (9.7%)	58 (16%)	870 (9.4%)	
More than high school	3,252 (67.6%)	120 (44%)	3,132 (69%)	
Marital status				**0.002**
Married/cohabiting	3,492 (71%)	134 (60%)	3,358 (71.4%)	
Never married	459 (6.4%)	36 (10%)	423 (6.2%)	
Widowed/divorced/separated	1,460 (22.6%)	102 (30%)	1,358 (22.4%)	
CVD	722 (11%)	109 (38%)	613 (9.3%)	**<0.001**
Hypertension	2,477 (40%)	173 (60%)	2,304 (39%)	**<0.001**
Diabetes	1,009 (14%)	76 (28%)	933 (13%)	**<0.001**
Physical activity				0.3
Active	1,572 (29%)	82 (33%)	1,490 (29%)	
Inactive	3,839 (71%)	190 (67%)	3,649 (71%)	
Total calories (Kcal/day)	2,035 (696)	1,929 (750)	2,041 (692)	0.084
CDAI	1.0 (3.6)	−0.4 (3.5)	1.0 (3.6)	**<0.001**
Vitamin A (ug/day)	650 (478)	531 (391)	656 (481)	**<0.001**
Vitamin C (mg/day)	80 (68)	70 (82)	81 (67)	0.12
Vitamin E (mg/day)	9.3 (5.6)	7.5 (4.4)	9.4 (5.6)	**<0.001**
Zinc (mg/day)	11.0 (5.2)	9.9 (4.8)	11.0 (5.2)	**0.005**
Selenium (ug/day)	113 (48)	103 (50)	114 (48)	**0.029**
Carotenoid (ug/day)	9,747 (9,567)	7,392 (8,534)	9,868 (9,602)	**0.001**

1 Mean ± SD for continuous; n (%) for categorical.

2 *t-test adapted to complex survey samples; chi-squared test with Rao & Scott’s second-order correction.*

PIR, poverty income ratio; BMI, body mass index; CVD, cardiovascular disease; CDAI, Comprehensive Dietary Antioxidant Index; Bold values indicate *p*-value < 0.05.

**Figure 2:**
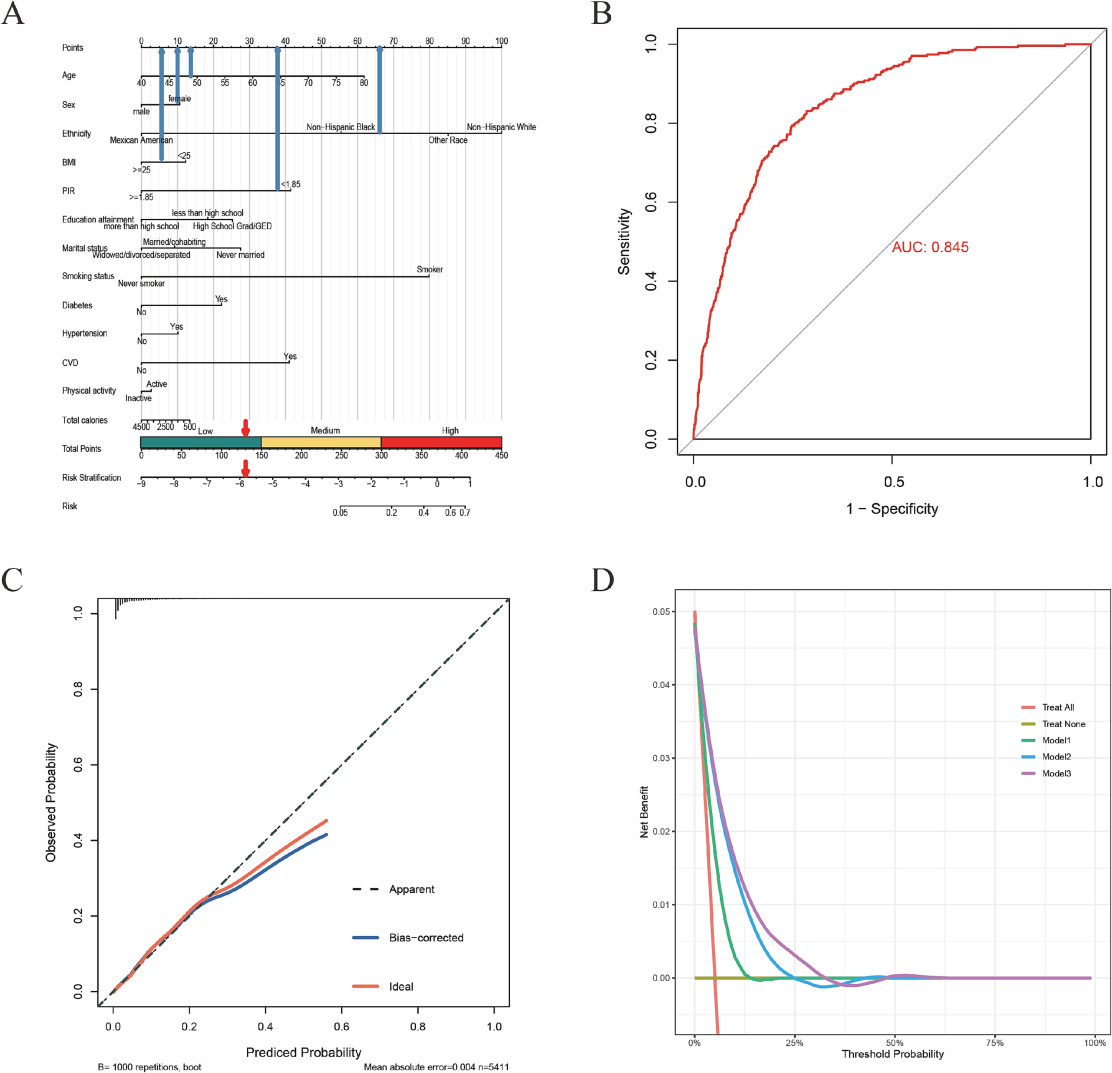
Nomogram and the validation of its ROC curve, calibration plot, and DCA. **(A)** Nomogram: Utilized for COPD risk assessment. **(B)** ROC Curve: The area under the ROC curve was employed to assess the accuracy of the nomogram. **(C)** Calibration Plot: Represents the diagnostic nomogram’s calibration, with the ideal prediction shown by the diagonal 45-degree line. **(D)** DCA: Provides an analysis of the net benefit derived by adding true positives and subtracting false positives, illustrating the effectiveness of the diagnostic nomogram in clinical decision-making; Modle 1: adjusted for age, sex, and ethnicity; Modle 2: adjusted for age, sex, ethnicity, PIR, BMI, education level, smoking status and physical activity; Modle 3: adjusted for age, sex, ethnicity, PIR, BMI, education level, smoking status, physical activity, total calories, cardiovascular disease, hypertension and diabetes.

Based on covariates, we constructed a COPD risk prediction nomogram (Figure 2A). The nomogram accurately delineates the impact of each factor on COPD. The ROC curve showed a high area under the curve (AUC) value: 0.845 (Figure 2B). By examining the calibration plot, the observed outcomes were highly consistent with the predicted results (Figure 2C). Additionally, DCA indicated that the nomogram model is effective in clinical practice (Figure 2D).

### Association between CDAI(including vitamin A, vitamin C, vitamin E, zinc, selenium, and carotenoids) and COPD

We initially used CDAI to assess the relationship between the overall antioxidant capacity of six dietary components and the risk of COPD. The results showed that in the fully adjusted model (Model 3), compared to the lowest quartile of CDAI, the highest quartile (Q4) was associated with a reduced risk of COPD (OR: 0.47, 95% CI: 0.22–0.97; Table 2). For the four quartiles of exposure to the six dietary antioxidants, Model 3 results demonstrated a negative correlation between dietary intake of vitamin A, vitamin C, vitamin E, zinc, selenium, and carotenoids with the risk of COPD (Table 2).

**Table 2:** Association of CDAI (including dietary vitamin A, vitamin C, vitamin E, zinc, selenium, and carotenoids) with the risk of COPD.

Quartiles	Model 1	Model 2	Model3
	OR (95% CI)	OR (95% CI)	OR (95% CI)
CDAI			
Q1	Reference	Reference	Reference
Q2	0.44(0.29, 0.66) ^***^	0.56(0.36, 0.87) ^*^	0.51(0.30, 0.87) ^*^
Q3	0.40(0.25, 0.66) ^***^	0.50(0.30, 0.82) ^**^	0.46(0.26, 0.82) ^*^
Q4	0.36(0.23, 0.56) ^***^	0.55(0.34, 0.89) ^*^	0.47(0.22, 0.97) ^*^
P for trend	*p* < 0.05	*p* < 0.05	*p* < 0.05
Vitamin A			
Q1	Reference	Reference	Reference
Q2	0.51(0.31, 0.87) ^*^	0.65(0.38, 1.09)	0.60(0.37, 0.99) ^*^
Q3	0.45(0.30, 0.67) ^***^	0.60(0.38, 0.94) ^*^	0.57(0.35, 0.93) ^*^
Q4	0.31(0.18, 0.53) ^***^	0.41(0.23, 0.74) ^**^	0.39(0.21, 0.73) ^**^
P for trend	*p* < 0.05	*p* < 0.05	*p* < 0.05
Vitamin C			
Q1	Reference	Reference	Reference
Q2	0.48(0.30, 0.76) ^**^	0.57(0.36, 0.91) ^*^	0.55(0.34, 0.90) ^*^
Q3	0.46(0.32, 0.68) ^***^	0.62(0.41, 0.93) ^*^	0.60(0.39, 0.92) ^*^
Q4	0.39(0.25, 0.63) ^***^	0.60(0.37, 0.97) ^*^	0.60(0.34, 1.08)
P for trend	*p* < 0.05	*p* = 0.06	*p* = 0.09
Vitamin E			
Q1	Reference	Reference	Reference
Q2	0.47(0.31, 0.73) ^***^	0.58(0.38, 0.88) ^*^	0.56(0.36, 0.88) ^*^
Q3	0.44(0.29, 0.67) ^***^	0.56(0.39, 0.83) ^**^	0.52(0.33, 0.82) ^**^
Q4	0.33(0.20, 0.54) ^***^	0.44(0.26, 0.75) ^**^	0.39(0.20, 0.76) ^**^
P for trend	*p* < 0.05	*p* < 0.05	*p* < 0.05
Zinc			
Q1	Reference	Reference	Reference
Q2	0.55(0.35, 0.85) ^**^	0.71(0.45, 1.13)	0.68(0.42, 1.12)
Q3	0.52(0.35, 0.77) ^**^	0.62(0.38, 1.01)	0.57(0.34, 0.95) ^*^
Q4	0.54(0.36, 0.81) ^**^	0.64(0.43, 0.97) ^*^	0.57(0.31, 1.03)
P for trend	*p* < 0.05	*p* = 0.12	*p* = 0.14
Selenium			
Q1	Reference	Reference	Reference
Q2	0.60(0.41, 0.89) ^*^	0.59(0.37, 0.94) ^*^	0.54(0.31, 0.95) ^*^
Q3	0.65(0.44, 0.95) ^*^	0.70(0.47, 1.04)	0.66(0.40, 1.10)
Q4	0.48(0.28, 0.83) ^*^	0.53(0.31, 0.91) ^*^	0.47(0.23, 0.95) ^*^
P for trend	*p* < 0.05	*p* < 0.05	*p* < 0.05
Carotenoids			
Q1	Reference	Reference	Reference
Q2	0.86(0.57, 1.30)	0.99(0.64, 1.55)	0.92(0.60, 1.43)
Q3	0.55(0.31, 0.97) ^*^	0.73(0.41, 1.30)	0.68(0.39, 1.18)
Q4	0.38(0.24, 0.59) ^***^	0.51(0.32, 0.80) ^**^	0.48(0.28, 0.82) ^**^
P for trend	*p* < 0.05	*p* < 0.05	*p* < 0.05

Modle 1: adjusted for age, sex, and ethnicity. Modle 2: adjusted for age, sex, ethnicity, PIR, BMI, education level, smoking status and physical activity. Modle 3: adjusted for age, sex, ethnicity, PIR, BMI, education level, smoking status, physical activity, total calories, cardiovascular disease, hypertension and diabetes.95% CI, 95% confidence interval; OR, odds ratio; CDAI, Comprehensive Dietary Antioxidant Index. *p < 0.05, **p < 0.01, ***p < 0.001; *p* < 0.05 was considered statistically significant.

Furthermore, we utilized a multivariate Cox regression model to assess the relationship between CDAI and all-cause mortality in COPD patients. In this cohort study, after excluding missing values, the median follow-up time was 38 months, involving a total of 271 COPD patients, of whom 47 (17.3%) died. CDAI was not significantly associated with all-cause mortality in COPD patients. However, among the six dietary antioxidants, Model 3 results showed that being in the second quartile (Q2) of zinc intake was associated with a reduced risk of all-cause mortality in COPD (HR: 0.25; 95% CI: 0.10–0.62) compared to the lowest quartile (Table 3). On the other hand, being in the third quartile (Q3) of vitamin E intake was associated with an increased risk of all-cause mortality in COPD (HR: 4.08, 95% CI: 1.40–11.9) compared to the lowest quartile (Table 3).

**Table 3:** Association between CDAI (including dietary vitamin A, vitamin C, vitamin E, zinc, selenium, and carotenoids) and all-cause mortality in COPD patients.

Quartiles	Model 1	Model 2	Model3
	HR (95% CI)	HR (95% CI)	HR (95% CI)
CDAI			
Q1	Reference	Reference	Reference
Q2	1.36(0.51, 3.65)	1.60(0.64, 3.97)	1.61(0.61, 4.23)
Q3	0.82(0.27, 2.47)	0.70(0.27, 1.81)	0.68(0.19, 2.42)
Q4	1.28(0.38, 4.37)	1.35(0.44, 4.13)	1.30(0.32, 5.27)
P for trend	*p* = 0.89	*p* = 0.97	*p* = 0.97
Vitamin A			
Q1	Reference	Reference	Reference
Q2	1.14(0.42, 3.12)	0.97(0.34, 2.74)	0.94(0.28, 3.12)
Q3	0.67(0.27, 1.66)	0.62(0.24, 1.61)	0.60(0.18, 2.02)
Q4	1.23(0.38, 3.93)	0.98(0.31, 3.06)	0.89(0.22, 3.68)
P for trend	*p* = 0.96	*p* = 0.77	*p* = 0.72
Vitamin C			
Q1	Reference	Reference	Reference
Q2	1.47(0.44, 4.97)	1.55(0.54, 4.46)	1.56(0.52, 4.66)
Q3	1.46(0.54, 3.99)	1.39(0.53, 3.62)	1.48(0.53, 4.13)
Q4	1.14(0.36, 3.56)	1.07(0.34, 3.35)	1.03(0.28, 3.70)
P for trend	*p* = 0.82	*p* = 0.96	*p* = 0.99
Vitamin E			
Q1	Reference	Reference	Reference
Q2	0.85(0.35, 2.07)	0.72(0.30, 1.76)	0.93(0.34, 2.59)
Q3	3.24(1.31, 8.02) ^*^	2.93(1.42, 6.06) ^**^	4.08(1.40, 11.9) ^**^
Q4	0.39(0.16, 0.98) ^*^	0.42(0.15, 1.16)	0.56(0.15, 2.06)
P for trend	*p* = 0.31	*p* = 0.43	*p* = 0.90
Zinc			
Q1	Reference	Reference	Reference
Q2	0.26(0.11, 0.65) ^**^	0.26(0.12, 0.56) ^***^	0.25(0.10, 0.62) ^**^
Q3	1.28(0.54, 3.04)	1.21(0.53, 2.79)	1.23(0.44, 3.43)
Q4	0.69(0.20, 2.34)	0.63(0.21, 1.86)	0.58(0.15, 2.29)
P for trend	*p* = 0.80	*p* = 0.92	*p* = 0.98
Selenium			
Q1	Reference	Reference	Reference
Q2	0.45(0.12, 1.70)	0.37(0.11, 1.23)	0.37(0.11, 1.29)
Q3	1.18(0.48, 2.89)	0.96(0.37, 2.47)	0.93(0.31, 2.77)
Q4	1.25(0.52, 3.04)	1.06(0.42, 2.68)	1.06(0.28, 3.99)
P for trend	*p* = 0.25	*p* = 0.45	*p* = 0.58
Carotenoids			
Q1	Reference	Reference	Reference
Q2	1.27(0.51, 3.19)	1.02(0.40, 2.64)	1.01(0.38, 2.73)
Q3	0.73(0.17, 3.08)	0.71(0.17, 3.00)	0.71(0.12, 4.06)
Q4	1.63(0.50, 5.35)	1.34(0.40, 4.43)	1.37(0.37, 5.12)
P for trend	*p* = 0.66	*p* = 0.81	*p* = 0.80

Modle 1: adjusted for age, sex, and ethnicity. Modle 2: adjusted for age, sex, ethnicity, PIR, BMI, education level, smoking status and physical activity. Modle 3: adjusted for age, sex, ethnicity, PIR, BMI, education level, smoking status, physical activity, total calories, cardiovascular disease, hypertension and diabetes. 95% CI, 95% confidence interval; HR, hazard ratio; CDAI, Comprehensive Dietary Antioxidant Index. *p < 0.05, **p < 0.01, ***p < 0.001; *p* < 0.05 was considered statistically significant.

### Nonlinear relationship assessment

**Figure 3:**
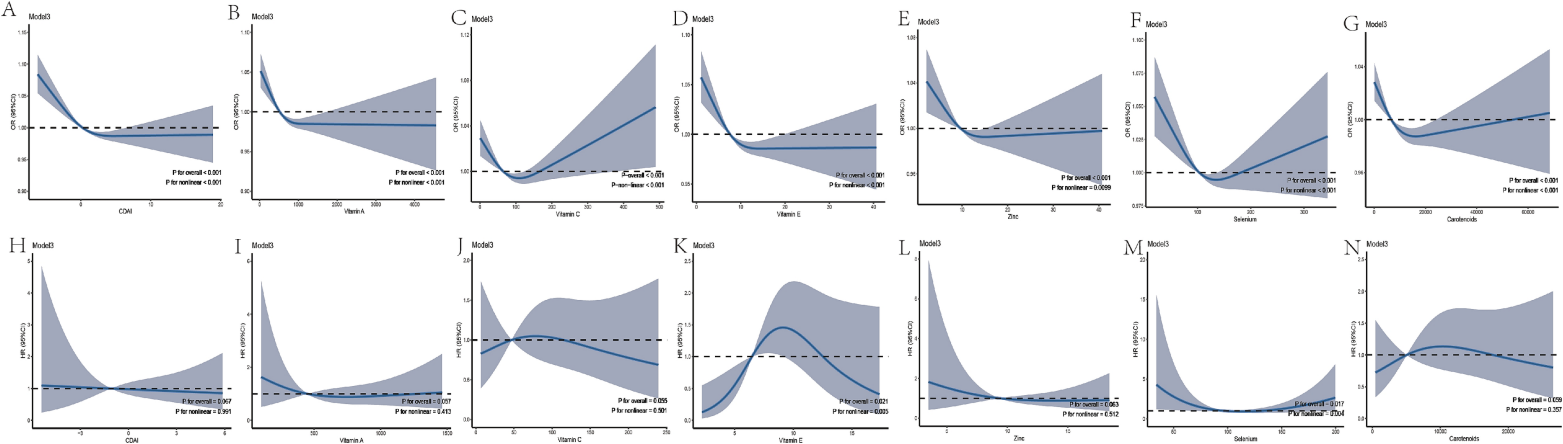
Restricted Cubic Spline Plots. Panels **A–G** show the relationship between CDAI, vitamin A, vitamin C, vitamin E, zinc, selenium, and carotenoids with COPD risk. Panels **H–N** display the relationship between CDAI, vitamin A, vitamin C, vitamin E, zinc, selenium, and carotenoids with all-cause mortality in COPD patients. Modle 3: adjusted for age, sex, ethnicity, PIR, BMI, education level, smoking status, physical activity, total calories, cardiovascular disease, hypertension and diabetes.95% CI, 95% confidence interval; OR, odds ratio; HR, hazard ratio; CDAI, Comprehensive Dietary Antioxidant Index.

To further explore the nonlinear associations between the CDAI (including dietary vitamin A, vitamin C, vitamin E, zinc, selenium, and carotenoids) and the risk of COPD and all-cause mortality, we conducted a RCS analysis based on the multivariate regression model (Model 3). The results showed that there were nonlinear associations between CDAI and the dietary intake of vitamin A, vitamin C, vitamin E, zinc, selenium, and carotenoids with the risk of COPD (all *p* for nonlinear < 0.05; Figures 3A–G). Regarding all-cause mortality, the results indicated an inverted “U”-shaped association between dietary vitamin E and all-cause mortality (*p* for nonlinear < 0.05), as well as a non-linear relationship between selenium and all-cause mortality. There is no non-linear relationship between CDAI and the dietary intake of vitamins A, C, zinc, and carotenoids with all-cause mortality (Figures 3H–N).

### Stratified and sensitivity analyses

**Figure 4:**
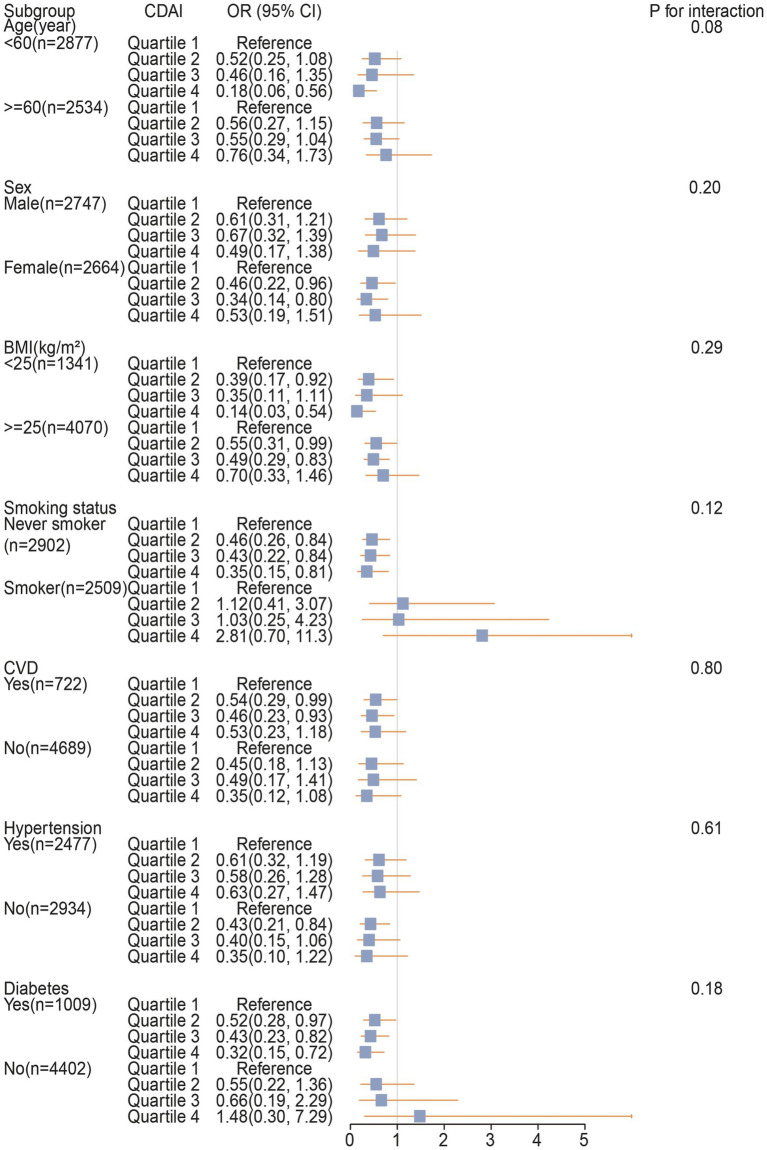
The association between CDAI and COPD in different subgroups. 95% CI, 95% confidence interval; OR, odds ratio; CDAI, Comprehensive Dietary Antioxidant Index; BMI, body mass index; CVD, cardiovascular disease.

The subgroup analysis results in Figure 4 indicate that the association between CDAI and COPD is more pronounced in individuals aged <60 years, females, never smokers, those without a history of hypertension, and patients with a history of CVD and diabetes. Additionally, no significant interactions were observed between CDAI levels and the stratified variables, with all interaction *p*-values exceeding 0.05.

In the sensitivity analysis, after excluding subjects who died within the last 2 years of follow-up, the results remained overall robust (Supplementary Table S1). When repeating the main analysis by quintiles of CDAI (including dietary vitamin A, vitamin C, vitamin E, zinc, selenium, and carotenoids), the association between selenium and the risk of COPD became non-significant, while the remaining associations did not show substantial changes (Supplementary Table S2). After additional adjustments for childhood asthma, vitamin A, vitamin E, zinc, and carotenoids were consistently associated with a lower risk of COPD (Supplementary Table S3).

## Discussion

In this study, we assessed the independent and joint associations between CDAI and COPD based on a nationally representative sample of the US population. After adjusting for all covariates, we observed a negative correlation between CDAI (including vitamin A, C, E, zinc, selenium, and carotenoids) and the risk of COPD. RCS demonstrated a nonlinear relationship between CDAI and COPD risk. Furthermore, we identified that compared to the lowest quartile, dietary zinc intake in the second quartile was inversely associated with the all-cause mortality risk in COPD patients, while dietary vitamin E intake in the third quartile was positively associated with the all-cause mortality risk.

Currently, there is scarce research on the association between CDAI and COPD. However, previous research on individual dietary antioxidants within the CDAI components (including vitamin A, vitamin C, vitamin E, zinc, selenium, and carotenoids) in relation to COPD further supports the findings of this study. In a cross-sectional study by Shen et al., vitamin A was suggested to reduce the risk of emphysema (22). Vitamin C, extensively studied for its antioxidant mechanisms, primarily contributes to cellular and extracellular water-soluble antioxidant capacity by scavenging reactive oxygen species (23). A systematic review by Lei (24) indicated that increased vitamin C intake may improve symptoms associated with COPD. Liu Z’s cross-sectional study suggested a reduction in COPD risk with increased vitamin E intake (18). Zinc deficiency not only impacts lung development but may also lead to lifelong impairment of lung function; therefore, maintaining zinc homeostasis is crucial for the stability of lung function (25). Moreover, a moderate increase in zinc concentration can activate ERK1/2-mediated autophagy, remove peroxides, and downregulate excessive inflammatory responses (25, 26), which holds significant importance for improving the condition of COPD patients and delaying disease progression (27). Lutein, a fat-soluble carotenoid, was significantly correlated with a lower FEV1/FVC% in current smokers (28). Animal experiments have shown that lycopene, a carotenoid, can counteract tobacco-induced COPD (29, 30). Currently, there is no cross-sectional study on dietary selenium intake and COPD. Our results suggest an inverse association between COPD risk and dietary selenium, specifically at the second quartile compared to the lowest quartile. Therefore, individual dietary antioxidants have independent effects on reducing COPD risk. However, the combined associations among dietary antioxidants should also be considered. We utilized CDAI to assess the combined intake of six dietary antioxidants to provide a more comprehensive perspective. Dietary regulation, as an external factor, involving the consumption of nutrients with antioxidant properties in a balanced dietary pattern, contributes to maintaining a stable redox state and counteracting oxidative stress effects (31). Our findings reveal a negative correlation between CDAI levels and COPD risk, consistent with previous studies on individual dietary antioxidants. However, of greater significance is the observed “U”-shaped non-linear relationship between CDAI and COPD risk, emphasizing the necessity for a balanced dietary model to derive greater benefits from the supplementation of dietary antioxidants.

We explored for the first time the association between CDAI (including vitamin A, vitamin C, vitamin E, zinc, selenium, and carotenoids) and all-cause mortality in a US population with COPD, using RCS analysis to investigate its non-linear relationship, and conducting sensitivity analysis by excluding follow-ups within the past 2 years. Our study suggests no association between CDAI and all-cause mortality among COPD patients. Nevertheless, zinc intake among individual dietary antioxidants is negatively correlated with all-cause mortality among COPD patients. Specifically, compared to the lowest quartile, being in the second quartile (Q2) of zinc intake is associated with reduced all-cause mortality among COPD patients. Previous studies have indicated that insufficient dietary zinc intake is common among COPD patients (32, 33). Hamon et al. measured zinc concentrations in bronchoalveolar lavage fluid of COPD patients and found significantly reduced zinc concentrations (34). On the other hand, low zinc levels may promote apoptosis of systemic endothelial cells and respiratory epithelial cells, associated with risks of airflow obstruction, oxidative stress, inflammation, and apoptosis (35–37). However, high zinc levels may induce apoptosis in pulmonary endothelial cells and exhibit cytotoxicity (27, 38), while also increasing reactive oxygen species production, leading to pulmonary oxidative stress (39). Therefore, in conjunction with our findings, zinc should be regarded as a “double-edged sword” in the body, suggesting that moderate rather than excessive zinc intake may be beneficial for COPD patients. Additionally, we observed a non-linear inverted “U”-shaped relationship between dietary vitamin E intake and all-cause mortality, similar to findings from a previous meta-analysis (40). This suggests that individuals with COPD should be cautious about the dosage of dietary vitamin E intake, as high doses of vitamin E may have pro-oxidant effects (41). Additionally, high doses of vitamin E might replace other fat-soluble antioxidants, such as gamma-tocopherol (42), disrupting the natural balance of the antioxidant system and increasing sensitivity to oxidative damage. In summary, the regulation of oxidative stress may play a crucial role in the prognosis and outcomes of COPD, and researchers still need to elucidate the specific mechanisms involved.

In subgroup analyses, we observed that in smoking individuals, despite higher levels of CDAI, there was no apparent risk reduction phenomenon. The specific reasons for this phenomenon are currently unclear, but it is likely associated with the higher levels of oxidative stress inherent in the smoking population (3). Furthermore, smoking may exacerbate oxidative burden by reducing endogenous antioxidant levels (43), thereby diminishing the protective effects of exogenous antioxidants in smokers (44). It is noteworthy that despite these considerations, the factor of smoking did not influence the conclusions of this study.

Given the increasing global burden of COPD, it is crucial to identify modifiable risk factors for the prevention and treatment of COPD. Our study analyzed both individual antioxidants and combined antioxidant effects, revealing that they have both independent and interrelated associations, and suggests that antioxidant intake may help reduce the risk of COPD and all-cause mortality. Therefore, future research should consider antioxidant therapy when designing preventive nutritional measures and further determine the optimal dosage and combinations of antioxidants to maximize their benefits. This may require further randomized controlled trials or long-term observational studies to achieve this goal.

We acknowledge limitations in our study. Firstly, the nature of NHANES data collection is essentially cross-sectional, making it challenging to establish a temporal relationship between the intake of various dietary antioxidants and the incidence of COPD. Secondly, the population with COPD was defined through self-reporting, which may introduce recall bias. However, these patients typically have a medical history and are informed about their COPD diagnosis by healthcare professionals. Additionally, despite adjustments for potential confounding factors, residual confounding may still be present. Furthermore, we conducted an association study using a nationally representative sample of the US population, and the observed associations may not be generalizable to other populations. Additional studies are required to ascertain if the advantages of dietary antioxidants can be applicable to other populations.

## Conclusion

This cross-sectional study suggests a negative correlation between higher CDAI levels and the risk of COPD, while appropriate intake of dietary antioxidant zinc may reduce all-cause mortality in COPD. However, further prospective and experimental research is needed to validate this association and explore its underlying mechanisms.

## Data Availability

The original contributions presented in the study are included in the article/Supplementary material, further inquiries can be directed to the corresponding author.

## Footnotes

^1^ https://www.cdc.gov/nchs/data-linkage/mortality.htm
