# Supplementary material for: The independent and joint relationships between dietary antioxidant intake with risk of chronic obstructive pulmonary disease and all-cause mortality: insights from NHANES
Source: Front Public Health. 2025 Jan 29;12:1393119. doi: 10.3389/fpubh.2024.1393119 (PMC11813870; doi:10.3389/fpubh.2024.1393119)
Supplement: Supplementary file 1 [file Data_Sheet_1.pdf]

Supplementary Online Content

TableS1 The association between all-cause mortality of COPD and CDAI (including dietary vitamin A, vitamin C, vitamin E, zinc, selenium, and carotenoids), excluding participants who died within the last 2 years of follow-up.

TableS2 The association between CDAI (including dietary vitamin A, vitamin C, vitamin E, zinc, selenium, and carotenoids) and COPD under quintiles.

TableS3 The association between CDAI (including dietary vitamin A, vitamin C, vitamin E, zinc, selenium, and carotenoids) and COPD, with additional adjustment for childhood asthma.

TableS1 The association between all-cause mortality of COPD and CDAI (including dietary vitamin A, vitamin C, vitamin E, zinc, selenium, and carotenoids), excluding participants who died within the last 2 years of follow-up.

Quartiles	Model 1	Model 2	Model3
	HR (95% CI)	HR (95% CI)	HR (95% CI)
**CDAI**			
Q1	Reference	Reference	Reference
Q2	1.86(0.64, 5.42)	2.21(0.85, 5.75)	2.26(0.75, 6.80)
Q3	1.27(0.41, 3.93)	0.99(0.37, 2.67)	0.89(0.24, 3.24)
Q4	1.80(0.47, 6.93)	1.67(0.55, 5.04)	1.39(0.31, 6.10)
**Vitamin A**			
Q1	Reference	reference	Reference
Q2	1.20(0.41, 3.54)	0.95(0.30, 3.08)	0.88(0.19, 3.96)
Q3	0.75(0.30, 1.91)	0.68(0.25, 1.84)	0.66(0.13, 3.30)
Q4	1.42(0.41, 4.90)	0.89(0.27, 2.95)	0.69(0.12, 4.05)
**Vitamin C**			
Q1	Reference	Reference	Reference
Q2	1.80(0.44, 7.29)	1.72(0.51, 5.78)	1.25(0.32, 4.84)
Q3	2.32(0.79, 6.87)	2.18(0.76, 6.20)	2.24(0.74, 6.76)
Q4	1.68(0.48, 5.82)	1.51(0.45, 5.07)	1.17(0.28, 4.86)
**Vitamin E**			
Q1	Reference	Reference	Reference
Q2	1.11(0.43, 2.85)	0.92(0.36, 2.37)	1.00(0.27, 3.71)
Q3	4.67(1.77, 12.3) **	4.27(2.06, 8.88) ***	4.40(1.12, 17.3) *
Q4	0.33(0.09, 1.24)	0.33(0.08, 1.33)	0.36(0.06, 2.20)
**Zinc**			
Q1	Reference	Reference	Reference
Q2	0.29(0.11, 0.81) *	0.25(0.10, 0.64) **	0.22(0.07, 0.68) **
Q3	1.44(0.56, 3.74)	1.18(0.39, 3.54)	1.15(0.33, 4.09)
Q4	1.00(0.26, 3.74)	0.74(0.23, 2.37)	0.56(0.13, 2.31)
**Selenium**			
Q1	Reference	Reference	Reference
Q2	0.33(0.07, 1.51)	0.24(0.07, 0.80) *	0.21(0.07, 0.67) **
Q3	1.32(0.52, 3.36)	1.07(0.34, 3.38)	0.87(0.27, 2.75)
Q4	1.28(0.49, 3.32)	0.90(0.39, 2.08)	0.62(0.20, 1.89)
**Carotenoids**			
Q1	Reference	Reference	Reference
Q2	1.70(0.59, 4.92)	1.53(0.48, 4.91)	1.46(0.40, 5.34)
Q3	1.01(0.21, 4.91)	1.03(0.21, 4.99)	0.99(0.14, 7.07)
Q4	2.29(0.67, 7.82)	1.69(0.54, 5.29)	1.82(0.47, 7.06)

Modle1: adjusted for age, sex, and race.

Modle2: adjusted for age, sex, race, PIR, BMI, education level, smoking status and physical activity.

Modle3: adjusted for age, sex, race, PIR, BMI, education level, smoking status, physical activity, total calories, cardiovascular disease, hypertension and diabetes.

95% CI, 95% confidence interval; HR, hazard ratio; CDAI, Comprehensive Dietary Antioxidant Index.

* $P < 0.05$, ** $P < 0.01$, *** $P < 0.001$; $P < 0.05$ was considered statistically significant.

TableS2 The association between CDAI (including dietary vitamin A, vitamin C, vitamin E, zinc, selenium, and carotenoids) and COPD under quintiles.

Quartiles	Model 1	Model 2	Model3
	OR (95% CI)	OR (95% CI)	OR (95% CI)
**CDAI**			
Q1	Reference	Reference	Reference
Q2	0.67(0.44, 1.01)	0.86(0.56, 1.32)	0.75(0.50, 1.13)
Q3	0.39(0.23, 0.66) ***	0.53(0.32, 0.89) *	0.44(0.25, 0.77) **
Q4	0.32(0.19, 0.53) ***	0.42(0.25, 0.70) **	0.36(0.20, 0.65) **
Q5	0.41(0.25, 0.67) ***	0.64(0.38, 1.10)	0.50(0.24, 1.04)
**Vitamin A**			
Q1	Reference	Reference	Reference
Q2	0.58(0.35, 0.97) *	0.74(0.48, 1.15)	0.71(0.45, 1.12)
Q3	0.35(0.23, 0.54) ***	0.47(0.29, 0.74) **	0.46(0.29, 0.73) **
Q4	0.37(0.23, 0.60) ***	0.52(0.30, 0.91) *	0.49(0.27, 0.88)
Q5	0.26(0.15, 0.45) ***	0.36(0.20, 0.65) **	0.33(0.18, 0.63) **
**Vitamin C**			
Q1	Reference	Reference	Reference
Q2	0.46(0.28, 0.75) **	0.59(0.36, 0.96) *	0.55(0.32, 0.93) *
Q3	0.32(0.18, 0.57) ***	0.43(0.24, 0.76) **	0.45(0.25, 0.81) *
Q4	0.40(0.24, 0.65) ***	0.59(0.35, 0.98) *	0.55(0.33, 0.91) *
Q5	0.32(0.20, 0.53) ***	0.52(0.30, 0.91) *	0.51(0.27, 0.98) *
**Vitamin E**			
Q1	Reference	Reference	Reference
Q2	0.34(0.20, 0.57) ***	0.43(0.25, 0.75) **	0.40(0.23, 0.69) **
Q3	0.45(0.28, 0.73) **	0.56(0.35, 0.89) *	0.53(0.31, 0.91) *
Q4	0.37(0.23, 0.61) ***	0.48(0.29, 0.79) **	0.43(0.24, 0.77) **
Q5	0.29(0.17, 0.51) ***	0.41(0.22, 0.75) **	0.33(0.16, 0.69) **
**Zinc**			
Q1	Reference	Reference	Reference
Q2	0.67(0.44, 1.00)	0.79(0.54, 1.17)	0.81(0.55, 1.18)
Q3	0.57(0.38, 0.86) **	0.72(0.44, 1.17)	0.70(0.40, 1.22)
Q4	0.45(0.30, 0.68) ***	0.57(0.37, 0.87) *	0.55(0.31, 0.96) *
Q5	0.63(0.38, 1.05)	0.77(0.48, 1.23)	0.74(0.39, 1.40)
**Selenium**			
Q1	Reference	Reference	Reference
Q2	0.73(0.46, 1.16)	0.77(0.46, 1.27)	0.74(0.44, 1.26)
Q3	0.58(0.34, 0.97) *	0.59(0.32, 1.08)	0.61(0.31, 1.20)
Q4	0.49(0.29, 0.82) **	0.58(0.34, 0.99) *	0.59(0.32, 1.07)
Q5	0.55(0.30, 1.01)	0.65(0.36, 1.17)	0.65(0.30, 1.38)
**Carotenoids**			
Q1	Reference	Reference	Reference
Q2	0.73(0.46, 1.17)	0.88(0.55, 1.39)	0.83(0.53, 1.30)
Q3	0.76(0.47, 1.22)	1.01(0.58, 1.76)	0.91(0.54, 1.53)
Q4	0.29(0.16, 0.53) ***	0.42(0.23, 0.77) **	0.39(0.22, 0.71) **
Q5	0.36(0.22, 0.61) ***	0.50(0.31, 0.81) **	0.46(0.26, 0.82) *

Modle1: adjusted for age, sex, and race.

Modle2: adjusted for age, sex, race, PIR, BMI, education level, smoking status and physical activity.

Modle3: adjusted for age, sex, race, PIR, BMI, education level, smoking status, physical activity, total calories, cardiovascular disease, hypertension and diabetes.

95% CI, 95% confidence interval; OR, odds ratio; CDAI, Comprehensive Dietary Antioxidant Index.

* P < 0.05, ** P < 0.01, *** P < 0.001; P < 0.05 was considered statistically significant.

TableS3 The association between CDAI (including dietary vitamin A, vitamin C, vitamin E, zinc, selenium, and carotenoids) and COPD, with additional adjustment for childhood asthma.

Quartiles	Model 1	Model 2	Model3
	OR (95% CI)	OR (95% CI)	OR (95% CI)
**CDAI**			
Q1	Reference	Reference	Reference
Q2	0.25(0.05, 1.39)	0.50(0.09, 2.63)	0.18(0.01, 2.37)
Q3	0.32(0.06, 1.69)	0.44(0.08, 2.50)	0.48(0.04, 6.44)
Q4	0.10(0.02, 0.55) *	0.23(0.04, 1.39)	0.10(0.00, 125)
**Vitamin A**			
Q1	Reference	Reference	Reference
Q2	0.10(0.01, 0.62) *	0.18(0.04, 0.86) *	0.02(0.00, 0.91) *
Q3	0.76(0.16, 3.68)	0.80(0.21, 3.09)	1.19(0.03, 46.3)
Q4	0.17(0.02, 1.81)	0.15(0.02, 1.16)	0.05(0.00, 3.02)
**Vitamin C**			
Q1	Reference	Reference	Reference
Q2	0.56(0.11, 2.90)	0.59(0.17, 1.99)	23.3(0.37, 1479)
Q3	0.67(0.14, 3.29)	1.23(0.20, 7.65)	3.61(0.22, 60.3)
Q4	0.04(0.00, 0.43) **	0.06(0.01, 0.64) *	0.22(0.02, 2.88)
**Vitamin E**			
Q1	Reference	Reference	Reference
Q2	0.09(0.02, 0.52) **	0.16(0.03, 0.81) *	0.04(0.00, 0.68) *
Q3	0.02(0.00, 0.19) ***	0.06(0.01, 0.62) *	0.03(0.00, 1.44)
Q4	0.31(0.08, 1.15)	0.64(0.15, 2.69)	0.57(0.05, 6.36)
**Zinc**			
Q1	Reference	Reference	Reference
Q2	0.20(0.04, 1.09)	0.20(0.02, 2.07)	0.04(0.00, 0.48) *
Q3	0.10(0.04, 0.26) ***	0.12(0.03, 0.43) **	0.01(0.00, 0.23) **
Q4	0.07(0.02, 0.33) **	0.08(0.01, 0.61) *	0.01(0.00, 1.15)
**Selenium**			
Q1	Reference	Reference	Reference
Q2	0.71(0.17, 2.99)	0.62(0.10, 4.05)	0.40(0.02, 8.12)
Q3	0.54(0.13, 2.21)	0.68(0.11, 4.32)	5.41(0.33, 89.4)
Q4	0.09(0.01, 0.63) *	0.11(0.01, 1.50)	0.04(0.00, 10.3)
**Carotenoids**			
Q1	Reference	Reference	Reference
Q2	0.27(0.08, 0.91) *	0.32(0.09, 1.12)	0.06(0.00, 2.30)
Q3	0.20(0.03, 1.33)	0.24(0.03, 1.63)	0.03(0.00, 0.41) *
Q4	0.05(0.01, 0.40) **	0.07(0.01, 0.73) *	0.00(0.00, 0.54) **

Modle1: adjusted for age, sex, and race.

Modle2: adjusted for age, sex, race, PIR, BMI, education level, smoking status and physical activity.

Modle3: adjusted for age, sex, race, PIR, BMI, education level, smoking status, physical activity, total calories, cardiovascular disease, hypertension, diabetes and Childhood Asthma.

95% CI, 95% confidence interval; OR, odds ratio; CDAI, Comprehensive Dietary Antioxidant Index.

* $P < 0.05$, ** $P < 0.01$, *** $P < 0.001$; $P < 0.05$ was considered statistically significant.
